# Views of Family Medicine Trainees of a Teaching Hospital in Riyadh regarding their Hospital Rotations: A Qualitative Study

**DOI:** 10.12669/pjms.291.2458

**Published:** 2013

**Authors:** Aljohara M Alquaiz, Hamza M Abdulghani, Syed Irfan Karim, Riaz Qureshi

**Affiliations:** 1 Aljohara M. Alquaiz, MD, MSc, MRCGP, Associate Professor, Department of Family Medicine, King Saud University, Saudi Arabia.; 2 Hamza. M Abdulghani, MD, ABFM, FRCGP, KSU Medical Education Chair for Research & Development, King Saud University, Saudi Arabia.; 3 Syed Irfan Karim, MD, MCPS, MRCGP, Deputy Director Post Graduate Program Family Medicine, King Saud University, Saudi Arabia.; 4 Riaz Qureshi, FRCGP, Professor, Department of Family Medicine, King Saud University, Saudi Arabia.

**Keywords:** Family Medicine Trainee, Hospital Training, Qualitative study, Saudi Arabia

## Abstract

***Objective: ***To explore Family Medicine Trainees views regarding the hospital component of their Family Medicine (FM) training program.

***Methodology: ***This is a qualitative focus group discussion based study. Thirteen trainees, eight from final year of FM training program and five from third year of the same program participated in the study. The structure for discussion included a previously distributed and completed questionnaire that included three sections. The first section was evaluation of the satisfaction of trainees with the different hospital specialties rotations. The second section related to reasons for rating the different rotations as excellent and very good. The third section related to deficiencies in training for those rotations which received a score of 3-5. The items in the questionnaire were utilized in the focus group discussion. Two facilitators who were investigators facilitated the discussion. The data was qualitatively analyzed to identify emergent themes and subthemes that described the trainees’ views.

***Results: ***The trainees highlighted the following views: Teaching in the hospital component is not relevant to the needs of Family Medicine trainees. Duration of the hospital posts should be reviewed. Emphasis should be on out-patient clinics rather than in-patient. More emphasis must be given to procedural skills, minor surgery and teaching in clinical contexts.

***Conclusion: ***Hospital training component of the Family Medicine training program should be reviewed, as the structure and its implementation doesn’t reflect the views of trainees regarding its relevance to their day to day practice.

## Introduction

 There is a world-wide need for well trained family physicians, in order to improve the health outcome indicators in every country. The Kingdom of Saudi Arabia is no exception to this need.^1^

 Evaluation is an essential part of the educational process. Educational organizations require evaluation as part of their quality assurance procedures.^2^ The whole curriculum of Family Medicine training of Saudi Arabia has been reviewed at the Saudi Commission for Health Specialties (SCFHS)^3^ Scientific Board level, but nothing of note has been done for the evaluation of the hospital clinical rotation training part of the program. Little is known about hospital component of clinical rotation from the trainees’ perspective. A local study, conducted mainly on the medicine specialty clinical rotation found that the majority of trainees were not satisfied with this rotation. They were treated as service residents, rather than family medicine trainees.^4^ Similar findings were found in some regional and international studies on hospital rotations, with conclusions that postgraduate family medicine training programs need to be evaluated and implemented according to the residents views and perceptions.^5^^,^^6^

 This study was carried out to explore trainees views regarding the hospital components of the Family Medicine training program at King Khalid University Hospital, Riyadh, Saudi Arabia.

## Methodology

 This is a qualitative study using focus group discussions with postgraduate trainees and trainers in the Family Medicine Training Program. The convenient study sample was composed of thirteen trainees from the 3^rd^ and 4^th^ year of training and 10 trainers, who were invited to participate in the study voluntarily.

 A self administered questionnaire was distributed to the thirteen trainees. The questionnaire included three sections. The first section was evaluation of the satisfaction of trainees with the different hospital specialties rotations. Satisfaction was measured on a scale of 1-5. Excellent one and unsatisfactory five. The second section related to reasons for rating the different rotations as excellent and very good. The third section related to deficiencies in training for those rotations which received a score of 3-5. The items in the questionnaire were utilized in the focus group discussion. Two of the researchers acted as moderators and facilitated the discussion by asking open ended questions.

 Areas tackled included training in out-patient clinics and in-patient care; availability of different learning activities; benefits of the on-call duties; and team interaction with the trainees. All trainees were encouraged to answer questions on their hospital clinical rotations by recalling learning experiences activities.Both moderators kept notes during all the discussions, prompting trainees to be more specific when necessary. The discussion lasted two hours and was audio taped and transcribed accurately. 

 Audiotapes were transcribed verbatim by investigators prior to analysis. The first step of analysis was to become familiar with the data, “familiarization” stage of the analytic process of qualitative research.^7^ The other four steps of the analysis were, identifying a thematic framework, indexing, charting and mapping and interpretation.^8^ Sub- themes were identified and collected to generate the major themes.

 All compiled data from the focus group discussion were highlighted and categorized into general themes. The audio-taped and transcription data were further independently analyzed by the authors, then discussed, summarized and modified until a consensus was reached.

 The final analysis findings were presented to all participants for validation. The participants were advised to feel free to modify or suggest any appropriate changes. They made few suggestions and indicated that the results summary reflected the focus group discussion accurately.^9^

 The study was approved by the Ethical Research Committee, at the College of Medicine, King Saud University.

## Results

 Out of the thirteen trainees who participated in the study, ten were males and three were females. R-4 trainees were those who had completed their hospital-based rotations and were posted at PHC rotation and R-3 trainees were those who had just completed all hospital-based clinical rotations and were posted in Community Medicine rotation.

**Fig.1:**
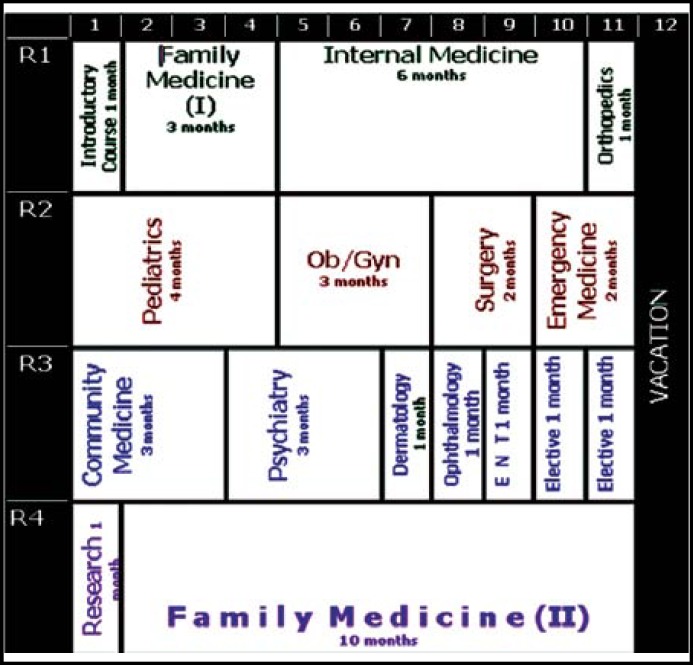
Four years Family Medicine Saudi Board Program Description

 The focus group discussion findings revealed issues, which were organized into four major themes: (1) teaching and training (2) relevance to the trainee’s needs (3) duration of training rotation and (4) educational environment. Table-I shows examples of trainees’ responses in each of the four themes. In the following text these four themes along with trainees’ quotations are illustrated as follows:

**Theme 1: Teaching and training:**

***A. Clinical teaching by trainers: ***The participants felt that there was lack of informal teaching during rounds, clinics and on-call duties.

 “ During surgical out-patient clinics there is no time for discussion or training for any procedural skills, like using proctoscope for diagnostic purposes or hemorrhoid ligation as a therapeutic procedure” [R3 Male # 9]

 They also felt that there was lack of supervision in Obstetrics and Gynecology (Obs/Gyn) and Accident and Emergency (A & E) rotations. 

*“During Obs/Gyn rotation, we were exposed to difficult and serious situations where we had to deal with patients without the supervision of the senior registrar who was usually busy”* [R4, female #10]*“Most of our duties were in the triage room with no senior registrar with us and no time for discussion”* [R3 Male # 9]

 There was no orientation of Family Medicine trainees at the beginning of new rotations. Majority of the trainees admitted that there was no orientation, when shifting from one post to another or from one specialty to another.

***B. Number of patients in clinics: ***The majority of the trainees agreed that the number of patients seen in the out-patient clinics run by trainees should be reduced to allow more time for discussion.

*“In the pediatric outpatient clinics we were working alone from the first day and seeing 25-30 patients /day without any senior registrar supervision”* [R3 male #5]

***C. Surgical procedures: ***The participants felt that they were not involved much in major and minor operations. There were very few surgical procedures which were performed by trainees: They also suggest that a list of operations, major and minor that need to be performed or attended by trainees should be developed.

 “I suggest that there should be one session /week only for procedures” [R3, male #6]

**Theme 2: Relevance to their needs:**

***A. Educational objectives: ***The participants felt that educational objectives should be updated, and should be specific for the learning needs of family medicine trainees.

***B. Irrelevant training for family physicians needs: ***Trainees were unsatisfied with the structure of the training, as they were mostly involved with inpatient work rather than outpatient clinics.

*“We had only one clinic/week in medicine post”* [R4, female #11]

*“There are very few out-patient clinics in surgery, I attended only two clinics in two months” *[R3 Male # 2]

Most of the trainees agreed that more outpatient clinical training is needed in all specialties.

 One trainee suggested, *“In medicine, I think that it is better to be in a general hospital, like Riyadh Medical Complex, which is a general hospital and get more training for outpatient clinics”* [R4 Male # 3]

 The group of trainees agreed that the six months medical rotation could be divided into three months in general medical ward and the other three months could be spent in out-patient clinics of medical subspecialty e.g. cardiology, rheumatology, hematology, endocrinology, pulmonology and gastroenterology and so forth .

 The participants also felt that it is important to specify the different needs of Family Medicine trainees to the different subspecialties in the medicine rotation.

*“In cardiology we were used as a filling gap, and my benefits were minimal*” [ R3 male # 3 ]

 Most of the trainees agreed that a change in the structure of ophthalmology and otolaryngology (ENT) rotations are required to include more primary care clinics, retinal clinics and one week attachment in emergency room.

*“there are very few and mostly complicated cases in the ENT /Ophthalmology posts that a primary care physician doesn’t need as a learning requirement and it is better to spend more time in their primary care clinics or emergency room”* [ R4 male # 6 ]

 Also they suggested that the distribution between the triage duties compared to the casualty duties should be 2:2 instead of 4:1. 

*“the load of patients in the triage room of Emergency Department is very high .Most interesting cases are managed in the casualty ,I wish we spent more time in the casualty”* [ R3 male # 9 ]

 One trainee suggested that one session /week should be reserved for clinical attachment in PHC centers during the three years of hospital rotation to expose trainee to the real world of primary care and how it relates to other specialties.

***C. Minor Surgery: ***There was a contradiction with the fact that they were required to attend major operations while they were not allowed to perform or attend minor surgery.

*“I attended every major operation done by my team but my benefit was zero”* [R3, male # 9]

**Theme 3: Duration of training rotation to be increased in some specialties:**

 The participants felt that the Ophthalmology, ENT and Dermatology rotations duration was short and a longer duration of at least two months was suggested.

*“Most of the ophthalmologists don’t believe in one month training and show no interest in teaching Family Medicine trainees”* [R4 male #3]

 One of the trainees suggested to increase the duration of Accident and Emergency rotation to three months instead of two months.

*“This rotation is very informative and rich of educational opportunities as we get in contact with emergency cases, I wish that the duration of this rotation is increased to three months”* [R4 ,male #7]

**Table-I:** Examples of trainees’ responses in each theme

*Themes*	*Example of trainees’ responses /Statements*
Teaching and training	Lack of informal teaching during rounds
	Lack of informal teaching during out-patient clinics
	Lack of informal teaching during on-calls
	Lack of supervision when dealing with difficult cases
	Lack of educational feedback
	Lack of learning procedure skills sessions
	Number of cases seen by trainees are too many in limited time.
	Trainees should be involved in major and minor operative procedures.
Relevance to their needs	Irrelevant training to my needs for being a family medicine trainee
	Little of out-patient and more in-patient clinics duties
	Little of general and more specialized clinic
	Little of primary care cases and more of complicated cases
	Little of minor surgery exposure and more time expended in
	complicated cases surgery
Duration of training rotation	Discrepancies in the required duration for different rotations
Educational environment	Attitude of consultants towards FM trainees makes them inferior
	Lack of appreciation from trainers

**Theme 4: Educational environment:**

 Attitudes of some consultants towards Family Medicine trainees were negative and there was lack of appreciation of their work.

 “*There is no appreciation of our efforts*” [R4, male #3]

*“I think it is important to involve all the consultants of specialties in the understanding of primary care, to encourage them to care and show interest in teaching and training of Family Medicine trainees and not to look at us as inferior to them, as was noticed by Family Medicine trainees in some of the departments”* [ R4, male #7]

**Fig.2:**
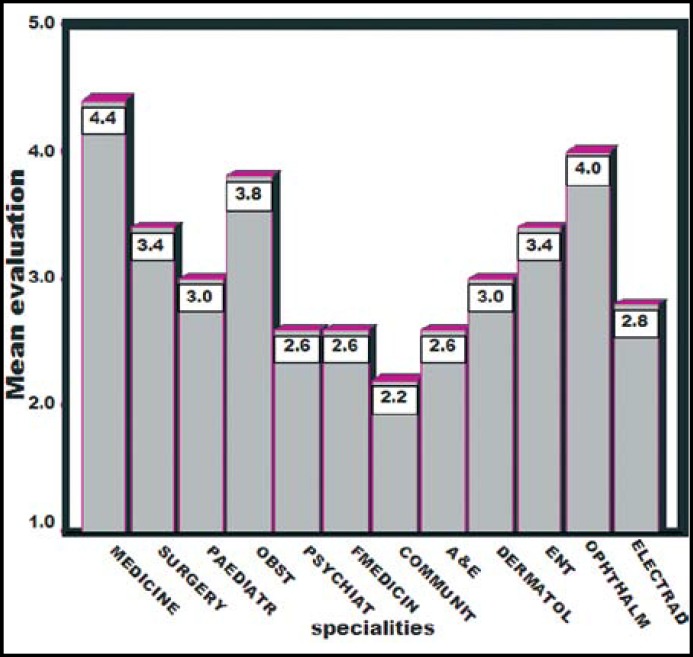
Evaluation of different specialties of hospital rotation.

1 = Excellent, 2 = V. Good, 3 = Good,

4 = Satisfactory, 5 = Unsatisfactory.

## Discussion

 The findings of this study clearly indicates that during hospital rotations, the educational needs of Family Medicine (FM) trainees were overlooked. The service requirements were generally high, which often overshadowed the learning requirements of the trainees. Prior international studies have also found similar impact of excessive service overload on trainees academic performance.^10^^,^^11^

 It was also pointed out that hospital based training gave no opportunity for experience in the community and FM trainees gained very little from the approach of consultants working in a hospital setting. Similar prior international studies have found that during in-patient rotations there is laborious demand of direct patient care without any direct supervision, which brings negative effect on the trainees’ performance.^12^ In addition, there is lack of close clinical supervision and availability of trainers in hospital rotations. Some trainees also felt that they were supervised by trainers who have no interest in clinical teaching.

 From interviews it became clear that Family Medicine trainees are assumed to have same training requirement as the specialty trainees in which they rotate by the hospital staff and faculty. Trainees also felt dissatisfied with the structure of their hospital training, in which they were mostly involved with in-patient services and were not exposed much to out-patient clinics. Other studies have also demonstrated that a lot of hospital based training has nothing to do with Family Medicine practice. It also appears that some health care professionals are not well informed about the nature and characteristics of family medicine training and its outcome.^13^ It has to be understood that Family Medicine trainees are not budding hospital doctors, rather they are budding family physicians. As a result of this attitude, when Family Medicine trainees come into real Family Practice, they may lack experience in the majority of the problems that could be relevant to Family Practice.^5^ Studies have also shown that during hospital rotation period, Family Medicine trainees should have clinical attachments in their Family Medicine clinics for at least one half day/week throughout the whole program.^4^

 On evaluation of the set of educational objectives, it appeared that many of the areas of rotations perceived to be most useful for FM trainees were not necessarily, addressed during their hospital rotations. The study participants were also concerned regarding insufficient number of out-patient clinics per week/trainee during their major hospital rotations in Medicine, Surgery, Pediatrics and Obstetrics & Gynecology. 

 Regarding the duration of specialty rotations in Dermatology, ENT and Eye, the trainees felt that they should be allowed to spend at least two to three months for gaining wider experience, which is quite similar to the perceptions of trainees in studies reported elsewhere.^14^

 This study highlights the need to focus on training in minor surgical procedures, especially during surgical rotations. Similar emphasis has been suggested in other studies, requiring an agreement with the surgical department on attaining of core procedural skills by the Family Medicine residents.^6^^,^^15^

 It was observed that the hospital specialists views on the subjects or topics which Family Medicine trainees require to master during hospital rotations are limited in their scope and inappropriate to Family practice setting. Likewise, the findings of this study and other qualitative studies have also highlighted the differences in the approach of hospital consultants and Family Medicine consultants to any clinical problem, especially while teaching Family Medicine trainees. Therefore, it is difficult for these hospital based consultants to be role models for Family Medicine trainees. These hospital consultants are not paid for their participation in Family Medicine trainees’ teaching. Regular teaching of Family Medicine residents by these consultants requires recognition of this responsibility in a consultants contract. It is recommended that these hospital consultants should be aware of Family Medicine curricular requirements and should work in conjunction with Family Medicine course coordinators.^16^ It has been suggested that Family Medicine trainers should also discuss the knowledge gained in hospitals in the context of primary health care and there should be some joint teaching seniors from both hospital consultants and family medicine trainers. This type of teaching is valued a lot by Family Medicine trainees, because they tend to have positive feelings for their trainers who not only focus on routine patient care, but also give more emphasis on trainees’ education.^17^

 The objectives for hospital rotations in the present SBFM curriculum manual have failed to delineate priorities for trainees in correlating their objectives to the learning context. The findings of this study indicate some basic problems in the curriculum, like making appropriate use of learning opportunities within hospital rotations, failure to identify core procedural skills for family medicine trainees and not being able to demonstrate that agreed skills have been acquired by the end of training.^2^ The Family Medicine trainees have grounds for suggesting that hospital component of SBFM training program should be re-structured and teaching should be improved. 

 The use of educational objectives in the hospital component of training could be beneficial for both FM trainers and hospital consultants. In a study of inpatient care and teaching, Hoffman and Donaldson found that all members of the team, both physicians and non physicians, learned from one another.^18^ Another study has shown that consultants, trainees and staff evaluation of teaching quality was influenced by the degree to which they were satisfied with their jobs.^19^ Therefore there should be trained teacher consultants with contractual time allocated for teaching. These consultants should also be aware of FM curriculum requirements and should work in conjunction with FM residency program coordinators. In order to keep the relevancy of their FM training throughout first 3 years of residency training the FM trainees should be in continuous contact with Family Medicine clinics (one clinic/week).^20^

 Applying these concepts, the program coordinators from other specialties should focus more on family medicine oriented teaching with protected time for reflection, assessment and feedback to trainees. It would definitely improve the quality of collegial relationship.^21^ There is also a need to include teaching skills program for Family Medicine trainees in the SBFM curriculum manual, which will enhance the role of being an effective future Trainer.^22^
